# A smart approach to EMG envelope extraction and powerful denoising for human–machine interfaces

**DOI:** 10.1038/s41598-023-33319-4

**Published:** 2023-05-12

**Authors:** Daniele Esposito, Jessica Centracchio, Paolo Bifulco, Emilio Andreozzi

**Affiliations:** Department of Electrical Engineering and Information Technologies, University of Naples Federico II, Via Claudio, 21, 80125 Naples, Italy

**Keywords:** Biomedical engineering, Data processing

## Abstract

Electromyography (EMG) is widely used in human–machine interfaces (HMIs) to measure muscle contraction by computing the EMG envelope. However, EMG is largely affected by powerline interference and motion artifacts. Boards that directly provide EMG envelope, without denoising the raw signal, are often unreliable and hinder HMIs performance. Sophisticated filtering provides high performance but is not viable when power and computational resources must be optimized. This study investigates the application of feed-forward comb (FFC) filters to remove both powerline interferences and motion artifacts from raw EMG. FFC filter and EMG envelope extractor can be implemented without computing any multiplication. This approach is particularly suitable for very low-cost, low-power platforms. The performance of the FFC filter was first demonstrated offline by corrupting clean EMG signals with powerline noise and motion artifacts. The correlation coefficients of the filtered signals envelopes and the true envelopes were greater than 0.98 and 0.94 for EMG corrupted by powerline noise and motion artifacts, respectively. Further tests on real, highly noisy EMG signals confirmed these achievements. Finally, the real-time operation of the proposed approach was successfully tested by implementation on a simple Arduino Uno board.

## Introduction

Electromyography (EMG)^1^ is one of the most used techniques to realize human–machine interfaces (HMIs)^2,3^ and assistive device control. EMG allows measuring the contraction of muscles by recording their electrical activity. This is usually performed via skin electrodes, so called surface EMG (sEMG). sEMG signal amplitude is a few millivolts and its power spectrum is mainly concentrated in 10–250 Hz band^1,4^. The EMG signal is rich in information, but only concise information on whether the muscle is active and/or the level of muscular contraction is usually of interest for HMI applications. To this aim, the EMG signal envelope is commonly used, rather than the raw signal. The combination of raw EMG signal rectification and low pass filtering is referred to as the EMG Linear Envelope (EMG-LE)^1^. The envelope of an EMG signal can also be estimated by computing the local root mean square (RMS) value of the raw EMG via a moving window. EMG-LE gives a measure of the local signal power and can be computed either via analog or digital processing^5^.

To date, many electronic boards are available for EMG signal acquisition, which can be interfaced with microcontroller boards to easily implement HMIs^2^. These EMG boards usually provide the EMG-LE to substantially reduce the required sampling rate (by at least one order of magnitude) compared to that required for acquisition of raw EMG signals (at least 1 kHz). However, sEMG signals are affected by various noises, the main ones being powerline interference (PLI) and motion artifacts, which can seriously impair the quality of the EMG-LE signals provided by such boards.

PLI is an electromagnetic interference that is caused by the capacitive or inductive coupling between EMG electrodes and the powerline in the surrounding environment^6–10^. In both cases, PLI makes a 50 Hz or 60 Hz sinewave (depending on the national regulations) and higher harmonics appear superimposed to the actual EMG signal. The PLI signal in EMG recordings can be much larger than the actual EMG signal, and its amplitude can vary over time.

Motion artifacts in EMG recordings are unavoidable, because they come from body motion, increase of muscle cross-section, and the resulting slippage of various tissues and skin layers^11^. The two main sources of motion artifacts in sEMG are the mechanical disturbance of the electrode charge layer, due to relative motion between the electrode and the underlying skin, and the deformation of the skin under the electrodes, which makes the potentials between skin layers change when skin is deformed or stretched^8,12^. The latter is much more difficult to attenuate, except by strongly abrading or puncturing the skin, which is not feasible in most practical cases.

**Figure 1:**
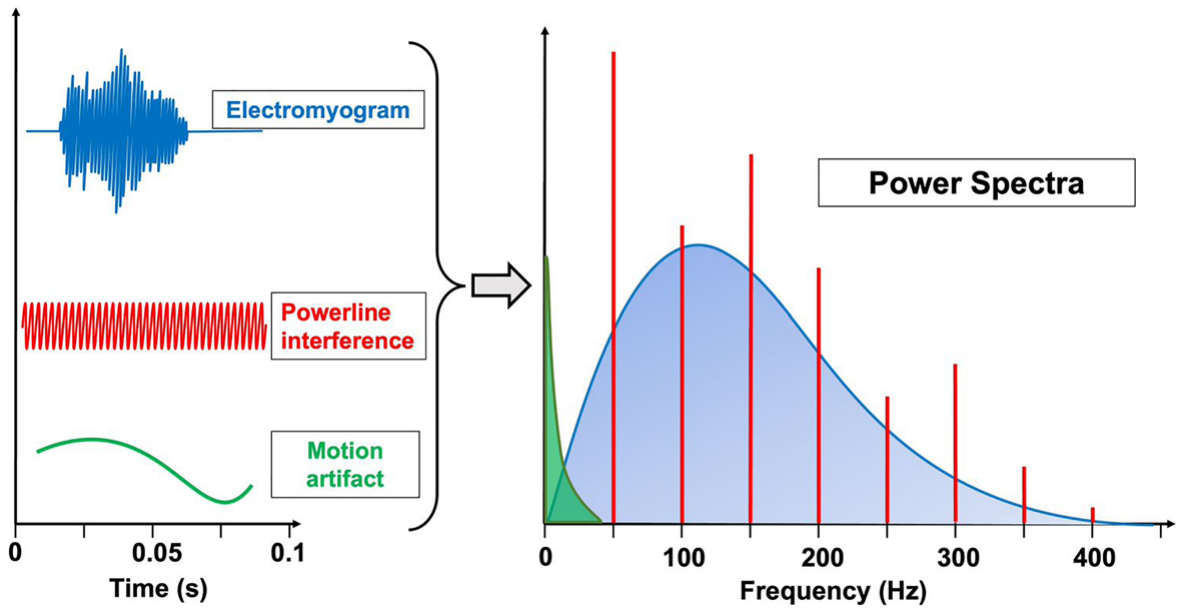
Qualitative sketch of sEMG signal, powerline interference and motion artifacts in time and frequency domains.

According to the International Society of Electrophysiology and Kinesiology standards, the frequency band of the diagnostic EMG signal ranges from 5–10 Hz up to 400–500 Hz^7^. Motion artifacts are generally confined to very low frequencies, typically below 20–30 Hz^1,13,14^. The spectrum of powerline interference should be concentrated at the powerline frequency (e.g., 60 Hz in Japan and almost all American continent, 50 Hz in Europe, Asia, Africa, Australia and some South American countries) but, due to non-linear loads increasingly used in modern equipment, higher harmonics of the fundamental powerline frequency are also present^8^. Figure 1 shows a qualitative sketch of sEMG and noise signals in the time domain, along with their power spectra in the frequency domain.

Most of the above-mentioned EMG boards providing EMG-LE signals directly, usually do not allow reducing the powerline interference nor the motion artifacts, which must be filtered before extracting the envelope, to obtain suitable EMG-LE signals. Consequently, these EMG boards often end-up providing noisy and uncertain envelope signals that cause malfunctioning of HMI applications, especially when the users are in environments with strong powerline electromagnetic fields, and when they perform large movements^15^. For this reason, some EMG boards also provide the raw EMG signal, in addition to the envelope signal.

In literature, many studies describe rather sophisticated methods to reduce EMG noises, which are usually based on digital filters^16,17^, neural networks (NN)^18^, wavelets^19^, ensemble empirical mode decomposition (EEMD)^20^, canonical correlation analysis (CCA)^21^, among many others. Many of these methods are currently not suitable for real-time operation. This is often due to heavy computational burdens that pose strong limitations, especially in the case of wearable or portable HMI applications, which are based on battery-powered devices. In fact, these devices are usually equipped with resource-limited computing platforms to achieve very low-power operation and thus cannot support demanding calculations. However, there has recently been an increasing interest in developing small neural networks, and in general processing algorithms that operate in real time, which are particularly optimized for embedded systems based on microcontrollers or programmable hardware (e.g., Field Programmable Gate Arrays, System-on-Chip), within the paradigm of edge computing.

This study presents a simple and elegant solution for real-time, noise free EMG-LE extraction with extremely low computational complexity. A feed-forward comb (FFC) filter^22^ removes both powerline interference and motion artifacts, while subsequent rectification and averaging provide the EMG-LE, without using a single multiplication.

Although the FFC filter is well-known in literature, its use for EMG denoising has never been investigated thoroughly. Probably, this has been due to the fact that the frequency response of the FFC filter causes substantial distortion to the EMG spectrum. Nevertheless, this study demonstrates that the FFC filter is effective for PLI and motion artifacts removal and preserves very well the morphology of the EMG-LE. Considering that this performance can be obtained at the cost of a single sum computation, as compared to other filtering approaches for noise removal, it follows that this solution offers great computational advantages for HMI applications. In fact, it can be easily implemented on very resource-constrained platforms developed for low-power applications, or used to save computational resources for more demanding tasks.

## Methods

### Feed-forward comb filter

The FFC filter operation is based on adding a delayed version of the input signal to itself, so as to produce constructive and destructive interference. The destructive interference mechanism is precisely that used to cut off a specific frequency component, along with its harmonics. The general difference equation of the FFC filter can be expressed as follows:

1 $$y\left(k\right)=x\left(k\right)+\alpha x\left(k-N\right),$$

where x is the input signal, N is the delay expressed in number of samples, and α is a parameter that regulates some aspects of the filter behaviour. In particular, when α is equal to ± 1, the minima of the FFC amplitude response are equal to zero, so they become nulls of the amplitude response. This choice is used to cancel out specific frequency components, such as the fundamental frequency of powerline interference and its harmonics.

The following equations report the expressions of the FFC amplitude responses for α = ± 1:

2 $$H\left({f}_{n}\right)=2\left|\mathrm{sin}(\pi N{f}_{n})\right|, \alpha =-1,$$

3 $$H\left({f}_{n}\right)=2\left|\mathrm{cos}(\pi N{f}_{n})\right|, \alpha =1,$$

where *f*_*n*_ is the normalized frequency, i.e. the absolute frequency *f* divided by the sampling frequency *f*_*s*_. The nulls of the amplitude responses in Eqs. (2) and (3) are reported in the following Eqs. (4) and (5), respectively:

4 $${f}_{null}=k\frac{{f}_{s}}{N}, k\in Z,$$

5 $${f}_{null}= \frac{{f}_{s}}{2N}+k\frac{{f}_{s}}{N}=\left(1+2k\right)\frac{{f}_{s}}{2N}, k\in Z.$$

The amplitude response in Eq. (2) has nulls for frequency values that are integer multiples of *f*_*s*_*/N*, while the amplitude response in Eq. (3) has nulls for odd integer multiples of *f*_*s*_*/(*2*N).* For this reason, the choice of α = − 1 is the one that allows removing the fundamental frequency of powerline noise interference and all its harmonics (both odd and even). Indeed, by choosing N equal to *f*_*s*_*/f*_*pli*_, with *f*_*pli*_ being the fundamental frequency to be cancelled, the nulls of the amplitude response would correspond to all integer multiples of *f*_*pli*_. On the contrary, by choosing α = 1, one would need to set N equal to *f*_*s*_*/(2f*_*pli*_*)* to make the first null to correspond to *f*_*pli*_ and all other nulls to odd multiples of *f*_*pli*_, which leaves even multiples of *f*_*pli*_ practically unaltered, thus resulting in poor PLI removal performances.

The difference equation of the specific FFC filter that was used in this study is reported below:

6 $$y\left(k\right)=x\left(k\right)-x\left(k-N\right).$$

**Figure 2:**
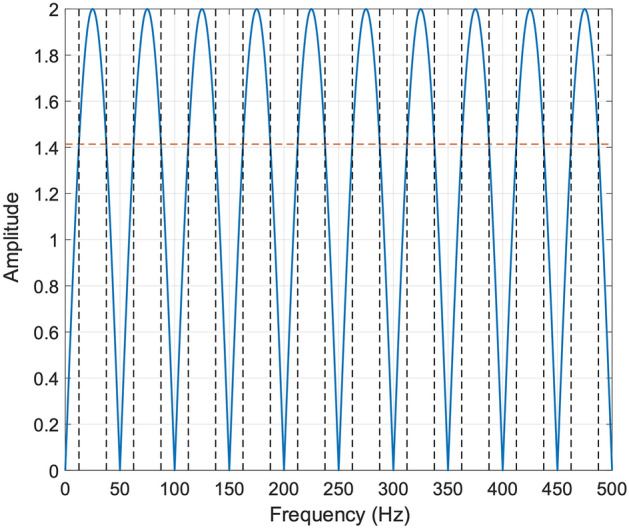
Amplitude response of the FFC filter with N = 20 and fs = 1000 Hz. The − 3 dB line is depicted as a red horizontal dashed line, while the black vertical dashed lines correspond to the cut-off frequencies.

Considering 50 Hz as the fundamental powerline interference frequency and assuming a sampling frequency of 1000 Hz, N must be set equal to 20. The amplitude response of the FFC filter with N = 20 is depicted in Fig. 2, where it could be observed that the nulls correspond to 0 Hz, 50 Hz and its integer multiples.

It is worth noting that the FFC filter has a zero DC gain with a high-pass behaviour at low frequencies. The − 3 dB cut-off frequencies can be computed as follows:

7 $$ \begin{gathered} \frac{{H(f_{{n3{\text{ dB}}}} )}}{{H_{{{\text{max}}}} }} = \frac{1}{\sqrt 2 }{ } \Rightarrow { }\left| {{\text{sin}}\left( {\pi Nf_{{n3{\text{ dB}}}} } \right)} \right| = \frac{1}{\sqrt 2 }{ } \Rightarrow { }f_{{n3{\text{ dB}}}} = \left\{ {\begin{array}{*{20}c} {\frac{1}{4N} + \frac{k}{N}} \\ {\frac{3}{4N} + \frac{k}{N}} \\ \end{array} } \right., \hfill \\ f_{{3{\text{ dB}}}} = \left\{ {\begin{array}{*{20}c} {\left( {\frac{1}{4} + k} \right)\frac{{f_{s} }}{N} = \left( {\frac{1}{4} + k} \right)f_{pli} = 12.5{\text{ Hz}} + k \cdot 50{\text{ Hz}}} \\ {\left( {\frac{3}{4} + k} \right)\frac{{f_{s} }}{N} = \left( {\frac{3}{4} + k} \right)f_{pli} = 37.5{\text{ Hz}} + k \cdot 50{\text{ Hz}}} \\ \end{array} } \right.. \hfill \\ \end{gathered} $$

**Figure 3:**
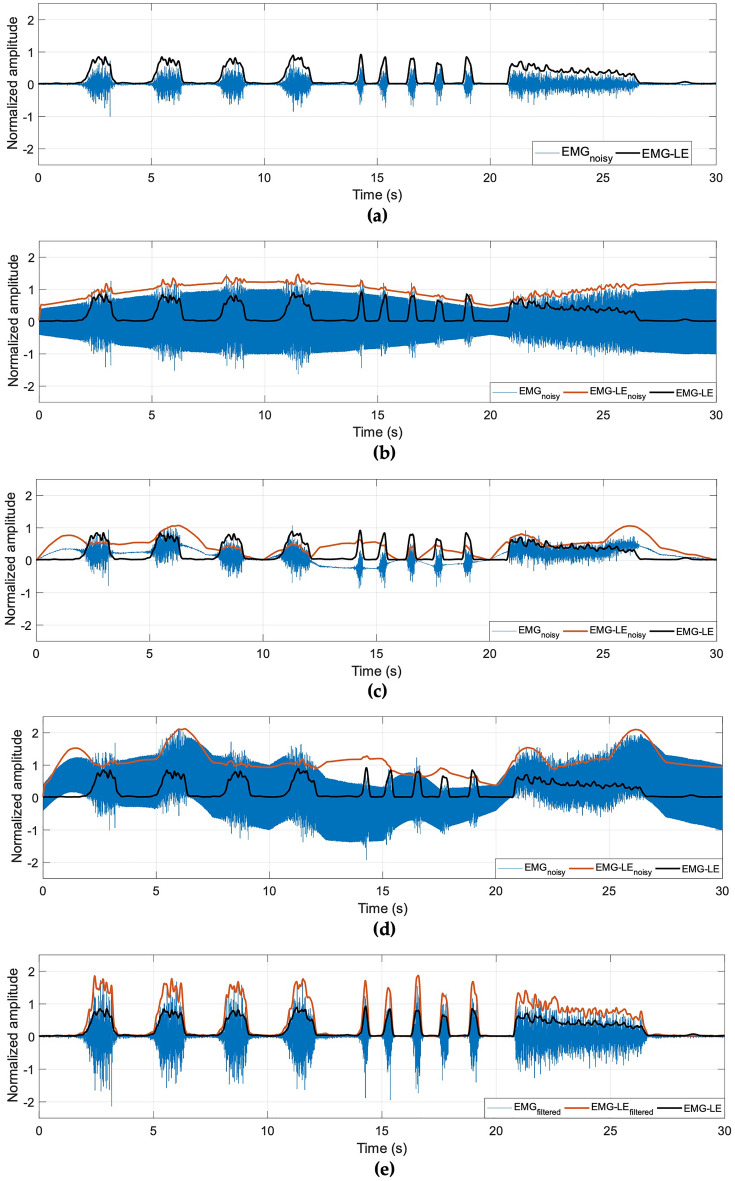
(**a**) Noiseless EMG signal with superimposed EMG-LE; EMG signal corrupted by (**b**) variable amplitude powerline interference, (**c**) baseline oscillations, (**d**) both, with corrupted EMG-LE and true EMG-LE; (**e**) EMG signal processed with FFC filter, recovered EMG-LE, true EMG-LE.

The FFC filter has two main effects: it operates as a comb filter for the removal of powerline interference and filters out the DC and near-DC components, where the power spectra of motion artifacts and baseline oscillations are mainly concentrated (below 20–30 Hz^4^). These aspects are very important for the extraction of the EMG-LE, which is usually computed by cascading a full-wave rectification (absolute value or power functions with even exponents) and a low-pass filtering (usually at 5-10 Hz for HMI applications)^3,7,23^. Indeed, both powerline interference and motion artifacts may severely corrupt the EMG-LE signal, and must be filtered out upstream of the actual envelope computation. Figure 3 shows an example of these issues (the EMG-LE signals were computed by cascading a full-wave rectification and a low-pass filtering at 5 Hz).

In most low-cost, low-power platforms for HMI implementation, a unipolar A/D converter is commonly used, so the digitized EMG signal exhibits only positive values and cannot be directly fed to the full-wave rectification stage^24,25^. In fact, the true reference voltage must first be determined and subtracted from the just digitized EMG, in order to recover the positive and negative values of the EMG. This is not always a straightforward operation, as unpredictable offset voltages may appear at the output of EMG measurement devices, which result in improper compensation of reference voltage. The zero DC gain of the FFC filter offers a simple and elegant solution to this problem, by providing a zero-mean EMG signal that is suitable for EMG-LE extraction.

It is worth noticing that the FFC filter rejects frequency bands of *f*_*pli*_/2 width, centered around the powerline interference components *k·f*_*pli*_, thus distorting the original EMG signal. However, it will be shown below that for the sole purpose of extracting the EMG-LE, such distortions are not of concern in practice.

In summary, the FFC filter has two main advantages: (a) it ensures the joint removal of powerline interference (with harmonics), offset voltages and motion artifacts/baseline oscillations, thus providing a pre-processed EMG signal that is suitable for EMG-LE extraction; (b) it has an extremely simple computation, that, in fact, requires only a single subtraction, thus resulting in an almost negligible computational burden even for resource-constrained platforms.

### Offline tests on noisy EMG signals

A quantitative assessment of the FFC filter performance was carried out by corrupting a noiseless real EMG signal with a simulated powerline interference, both with flat amplitude and with amplitude modulation. All signal processing operations were performed in MATLAB® R2017b (The MathWorks, Inc., 1 Apple Hill Drive, Natick, Massachusetts, 01760, USA). The noiseless EMG signal was extracted from a dataset acquired in a previous study^26^. No measurements were carried out during this study. The sEMG signals had been recorded at sampling frequency of 1 kHz from forearm muscles.

**Figure 4:**
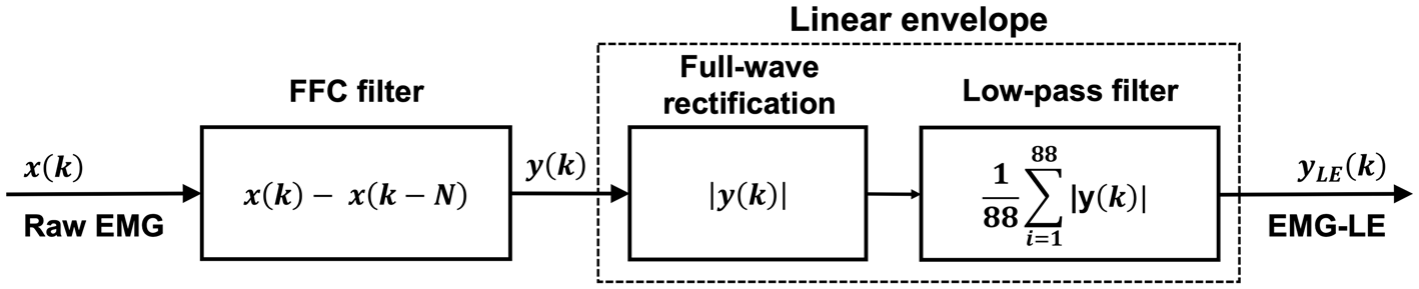
Block flow diagram of the various filtering stages applied on the raw EMG signal.

The powerline interference was simulated by using amplitudes and phases of the 50 Hz component and its harmonics up to 500 Hz, which had been extracted from a measurement of real powerline interference obtained with the same experimental set-up. An amplitude modulated powerline interference was also designed by using a slow sinewave as the modulating signal. The simulated noise was then added with increasing amplitudes to the noiseless EMG signal, so as to obtain SNR values between 0.05 and 10. The noisy EMG signals thus obtained were processed with the FFC filter. The linear envelopes of the noiseless, noisy and filtered EMG signals were computed by means of a full-wave rectification (absolute value operation) and a low-pass filtering at 5 Hz via a moving average (MA) on 88 samples (with a sampling frequency of 1000 Hz, a MA filter with N = 88 samples has a cut-off frequency of 5 Hz). The block diagram illustrating the various filtering stages is presented in Fig. 4.

Moreover, the cross-correlation between the noiseless EMG-LE signal and each noisy and filtered EMG-LE signal was computed by considering the whole length. Then, the maximum of the cross-correlation was located, the corresponding lag was used to re-align the signals, and the Pearson’s correlation coefficient was determined. The trends of the correlation coefficients with the SNR were finally compared.

A further quantitative assessment of the FFC filter performances was carried out by corrupting the same noiseless EMG signal with real motion artifacts. Signals chunks including only motion artifacts were extracted from the dataset^26^. The noiseless EMG signal was then corrupted by motion artifacts signals with increasing amplitudes, so as to obtain SNR values between 0.05 and 10. The corrupted EMG signals were processed with the FFC filter. Finally, the linear envelopes of the noiseless, corrupted and filtered EMG signals and the Pearson’s correlation coefficients between the noiseless EMG-LE signal and each corrupted and filtered EMG-LE signal were computed via the same procedures. The trends of the correlation coefficients with the SNR were finally compared.

Noisy EMG signals with powerline interference and motion artifacts were extracted from the same dataset to further test the performance of the FFC filter on actual noisy data.

### Performance comparison with other denoising algorithms

The performance of the FFC filter was compared with two denoising approaches proposed in literature^27,28^. The first approach operates in the frequency domain and mainly consists of Fast Fourier Transform (FFT) computation, cancellation of spectral coefficients corresponding to the harmonics of the powerline frequency and to the main spectral content of motion artifacts at low frequencies, and finally inverse FFT computation. This FFT-based approach was designed with the aim of reducing as much as possible the number of samples for FFT computation, to reduce the computational burden per sample in a sliding window application, or the computational delay in a block processing application. However, since the number of samples determines the frequency resolution of the FFT, a maximum resolution value of 25 Hz was set, which for EMG signals sampled at 1 kHz was achieved by computing the FFT on 40 samples, in order to be able to precisely cancel the spectral coefficients corresponding to the harmonics of the powerline frequency (50 Hz) and those corresponding to the 0–25 Hz range for motion artifacts removal. The second approach, referred to as FilterDxN, operates in the time domain and is essentially a high quality factor comb filter with integer coefficients, properly tuned to exhibit zeros at the harmonics of the powerline frequency. The FilterDxN features two parameters, namely N and D. As per indication of the original publication, N was set to 17, while D was set to the ratio of the sampling frequency (1000 Hz) and the powerline frequency (50 Hz), which in this study was equal to 20. Therefore, a Filter20x17 was actually considered for the tests. The two algorithms were tested both for PLI and motion artifacts removal, via the same methodologies adopted for the performance assessment of the FFC filter, previously described in the “Offline tests on noisy EMG signals” section. In particular, the tests on PLI with constant and modulated amplitude were performed by considering only the lowest SNR (0.05), because all algorithms showed stable performance at varying SNR. The performance on motion artifacts removal, instead, were assessed for all SNR values. Moreover, the computational and memory resources requirements were evaluated and compared for all the considered algorithms.

### Real-time tests on noisy EMG signals

The real time performance of the proposed approach was assessed via the following test scene. An arbitrary waveform generator (GW Instek AFG-2005, Good Will Instrument Co., Ltd., No.7-1, Jhongsing Road., Tucheng Dist., New Taipei City 236, Taiwan) was used to output voltage signals from real recordings of noisy EMG signals (previously acquired in Ref.^26^), so as to resemble a real scenario. The output voltage of the generator was fed to an off-the-shelf EMG sensor board “MyoWare Muscle Sensor”^29^, in order to amplify the EMG signals. The amplified signals, provided as an analog output on the “Raw EMG signal” pin of the “Myoware Muscle Sensor” board (pin 7), were fed to an analog input of an Arduino UNO board. At this stage, the raw EMG signals were first digitized at 1 kHz, using the on-board analog-to-digital converter (10-bit resolution), and then processed in real time via the proposed methodology. The open-source library TimerOne^30^ was used to ensure a precise sampling time. The EMG processing performed by the Arduino UNO board consisted of the FFC filtering for PLI and motion artifacts removal, signal rectification, and low-pass filtering at about 4 Hz, by using a MA filter with a window size of 128 samples to eventually obtain the EMG-LE. Each new sample of the EMG-LE was released at a rate of about 8 Hz and sent to a personal computer via a Bluetooth serial interface (HC-05^31^), connected to the Arduino UNO board. The full Arduino code is available as Supplementary Material.

It is worth noticing that other commercial EMG sensors, such as Seed EMG detector^24^ or Gravity EMG sensor^25^, do not offer a specific port for raw EMG output, rather providing only the EMG envelope. For this reason, such sensors are not compatible with the proposed methodology. The correct execution of the algorithm strictly requires a precise uniform sampling, ensured by the TimerOne library. Indeed, adding simple idle cycles to sample the raw EMG signal would result in nonuniform sampling and impair correct filter operation. In addition, by only changing the sampling frequency of the raw EMG signal (e.g., from 1000 to 1200 Hz), the algorithm could be easily adjusted for the removal of 60 Hz (and higher harmonics) powerline interference, as well as motion artifacts. Other parameters, such as the number of FFC filter coefficients (N = 20) and the width of the MA filter window (128 samples) for linear envelope computation, can remain unchanged. The use of a window size that is a power of 2 allows replacing the division required in the MA filter by a simple bit shifting, which has a negligible computational cost. Table 1 summarizes the algorithm specifications for 50 Hz or 60 Hz powerline frequencies.

**Table 1:** Parameters setting for 50 Hz or 60 Hz powerline interference.

	Parameters for 50 Hz PLI	Parameters for 60 Hz PLI
Raw EMG sampling frequency	1000 Hz	1200 Hz
Number of FFC filter coefficients	20	20
Lower cut-off frequency of FFC filter	≈ 12 Hz	≈ 15 Hz
Number of samples of MA filter window for EMG-LE computing	128	128
Upper cut-off frequency of MA filter for EMG-LE computing	≈ 4 Hz	≈ 5 Hz
Data output frequency from the Arduino Bluetooth serial port	≈ 8 Hz	≈ 9 Hz

## Results

### Offline tests on noisy EMG signals

**Figure 5:**
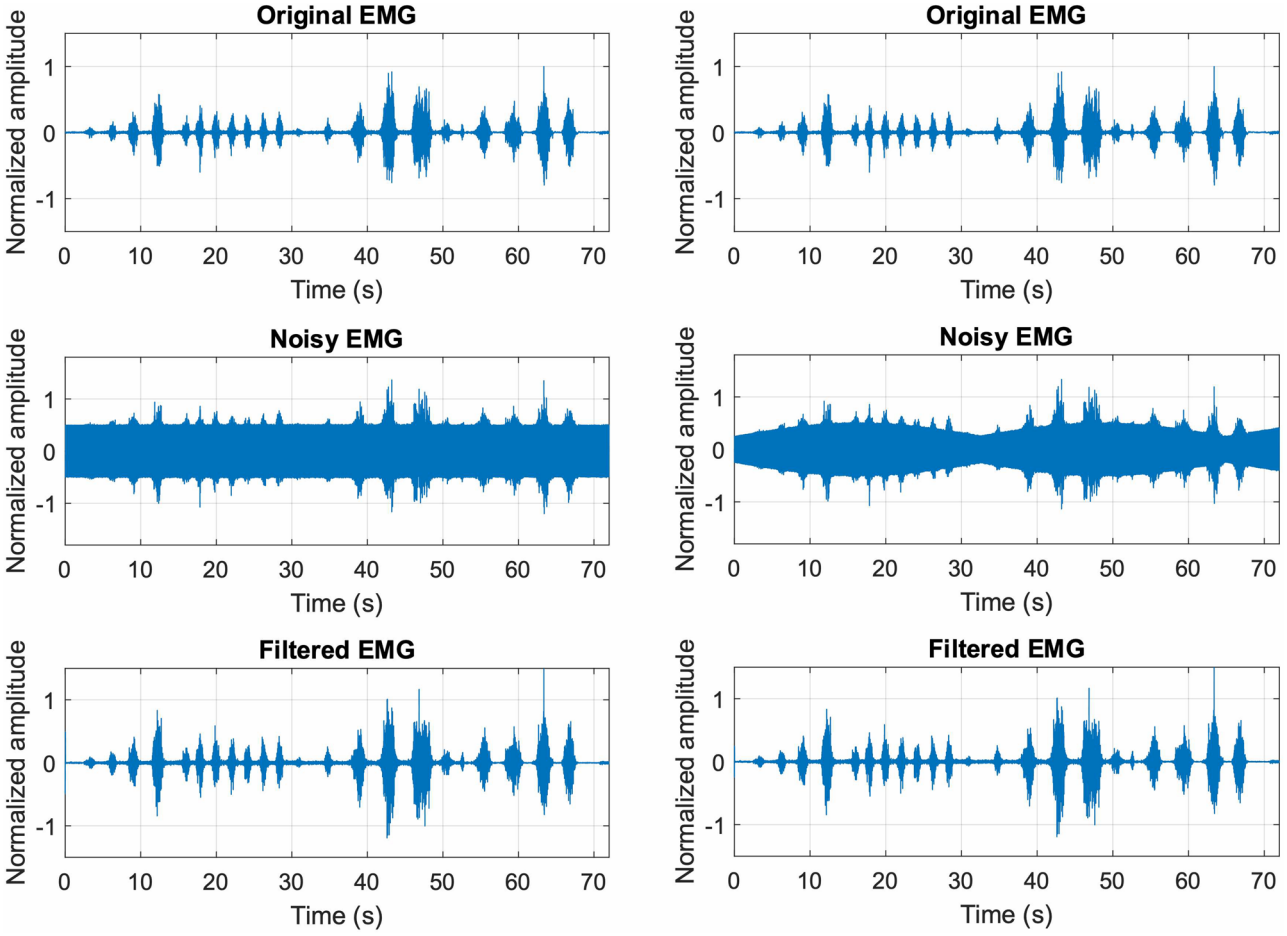
Top panels: noiseless EMG signal; central panels: noisy EMG signals obtained by corrupting the noiseless EMG signal with flat amplitude powerline noise (left) and amplitude modulated power-line noise (right); bottom panels: signals obtained by applying the FFC filter to the noisy EMG signal with flat amplitude (left) and amplitude modulated (right) powerline noise.

Figure 5 shows the noiseless EMG signal (top panels), the noisy signals (central panels) with SNR equal to 2 (both with flat amplitude and amplitude modulated noise) and the related filtered signals (bottom panels). It could be observed that the original EMG signals were efficiently recovered from both the very noisy EMG signals.

**Figure 6:**
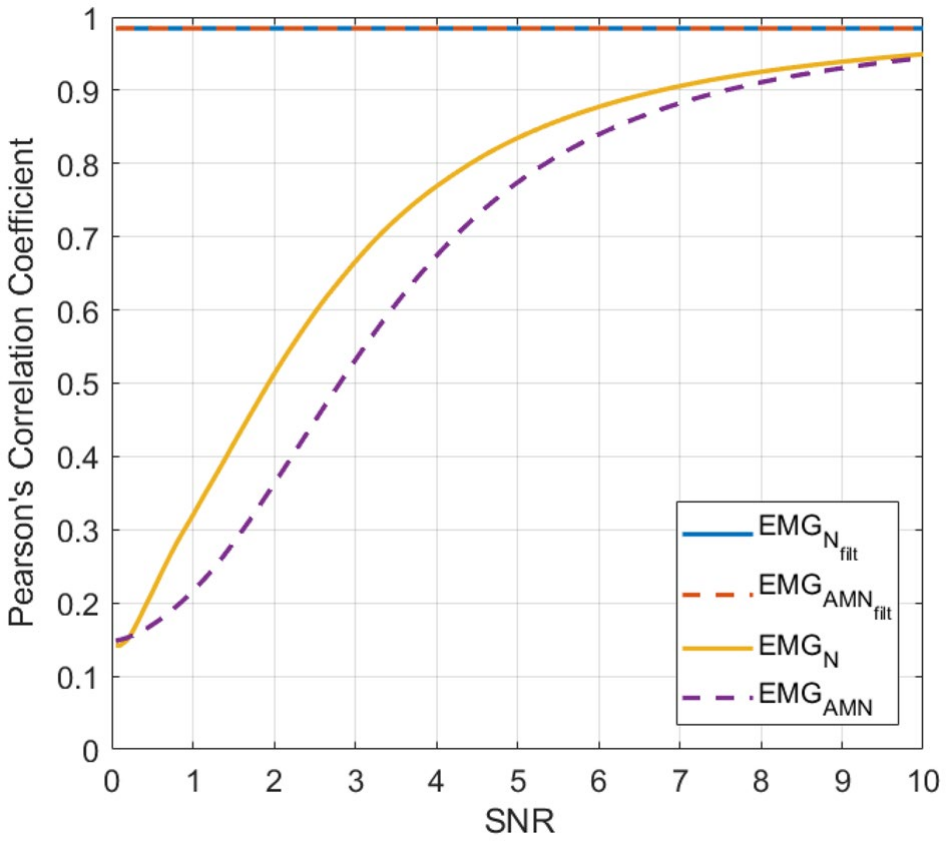
Pearson’s correlation coefficients of noisy and filtered EMG-LE with respect to noiseless EMG-LE. Solid lines refer to the EMG signal corrupted with flat amplitude powerline interference (yellow line) and the related filtered signal (blue line); dashed lines refer to the EMG signal corrupted with amplitude modulated powerline interference (purple line) and the related filtered signal (orange line).

This result is confirmed by the Pearson’s correlation coefficients scored by the filtered EMG-LE signals with respect to the corresponding noiseless EMG-LE signals, which turned out to be in excess of 0.98 for all values of the SNR (ranging from 0.05 to 10), as reported in Fig. 6. The same figure also depicts the correlation coefficients of the noisy EMG-LE with respect to the corresponding noiseless EMG-LE signals. It is worth noticing that for a SNR of 7, the correlation coefficients of the noisy signals already dropped below 0.9, and for a SNR of 2 (the same SNR value of the signals depicted in Fig. 5) correlation coefficients as low as 0.51 and 0.36 were obtained, respectively, for EMG signals corrupted by flat amplitude and amplitude modulated powerline interference.

**Figure 7:**
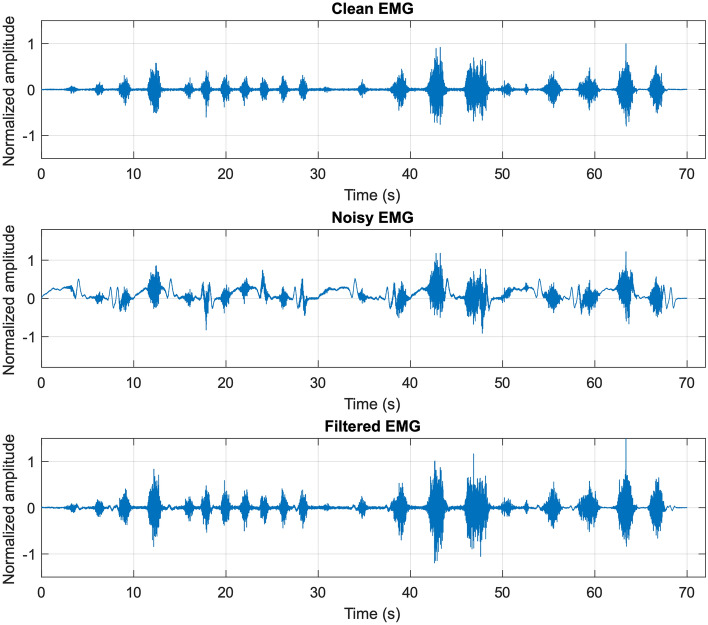
Top panel: noiseless EMG signal; central panel: EMG signal corrupted with motion artifacts (SNR = 2); bottom panel: filtered signal obtained by applying the FFC filter to the corrupted EMG signal depicted in the central panel.

**Figure 8:**
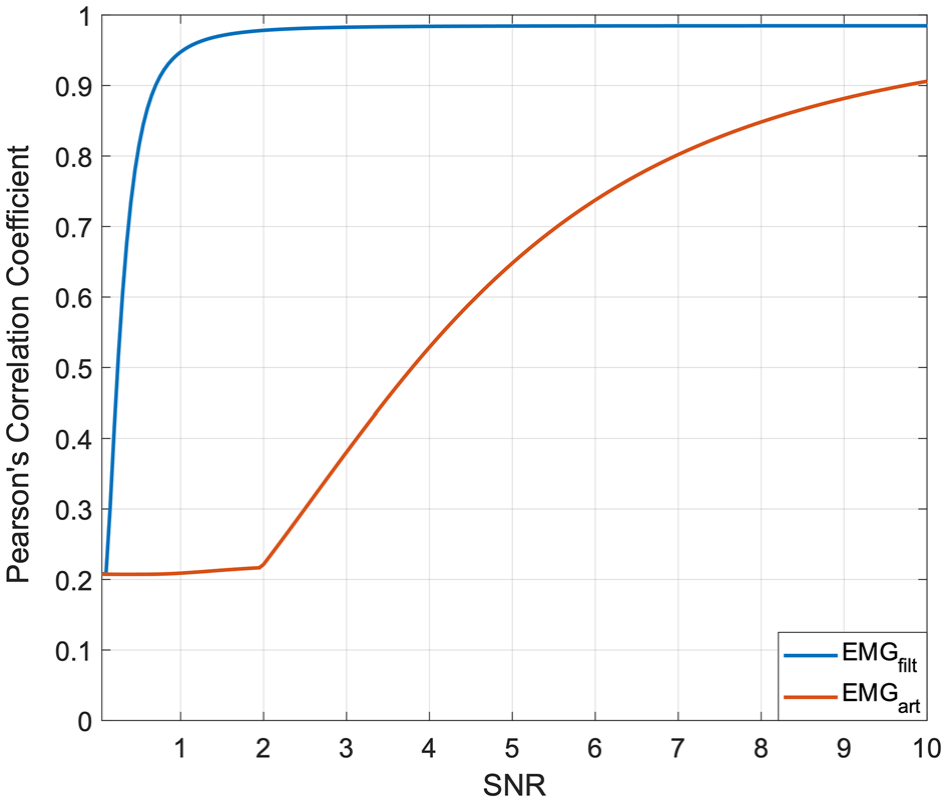
Pearson’s correlation coefficients of EMG-LE of signals corrupted by motion artifacts (red line) and related filtered signals (blue line) with respect to noiseless EMG-LE.

Figure 7 shows the noiseless EMG signal in the top panel, the EMG signal corrupted by motion artifacts with SNR equal to 2 in the central panel, and the related filtered signal in the bottom panel. Once again, the FFC filter successfully recovered the original EMG signal from its corrupted version. Indeed, the FFC filter provided Pearson’s correlation coefficients in excess of 0.94 for SNR values ranging from 1 to 10 (see Fig. 8). For lower SNR values, the correlation coefficients exhibited a rather brisk drop, while still maintaining a correlation gain in excess of 2 for SNR values down to 0.2. Regarding the corrupted EMG signals, it could be observed that for a SNR of 7, the correlation coefficients already dropped below 0.8, and for a SNR of 2 (the same SNR value of the signals depicted in Fig. 7) a correlation coefficient as low as 0.22 was obtained.

**Figure 9:**
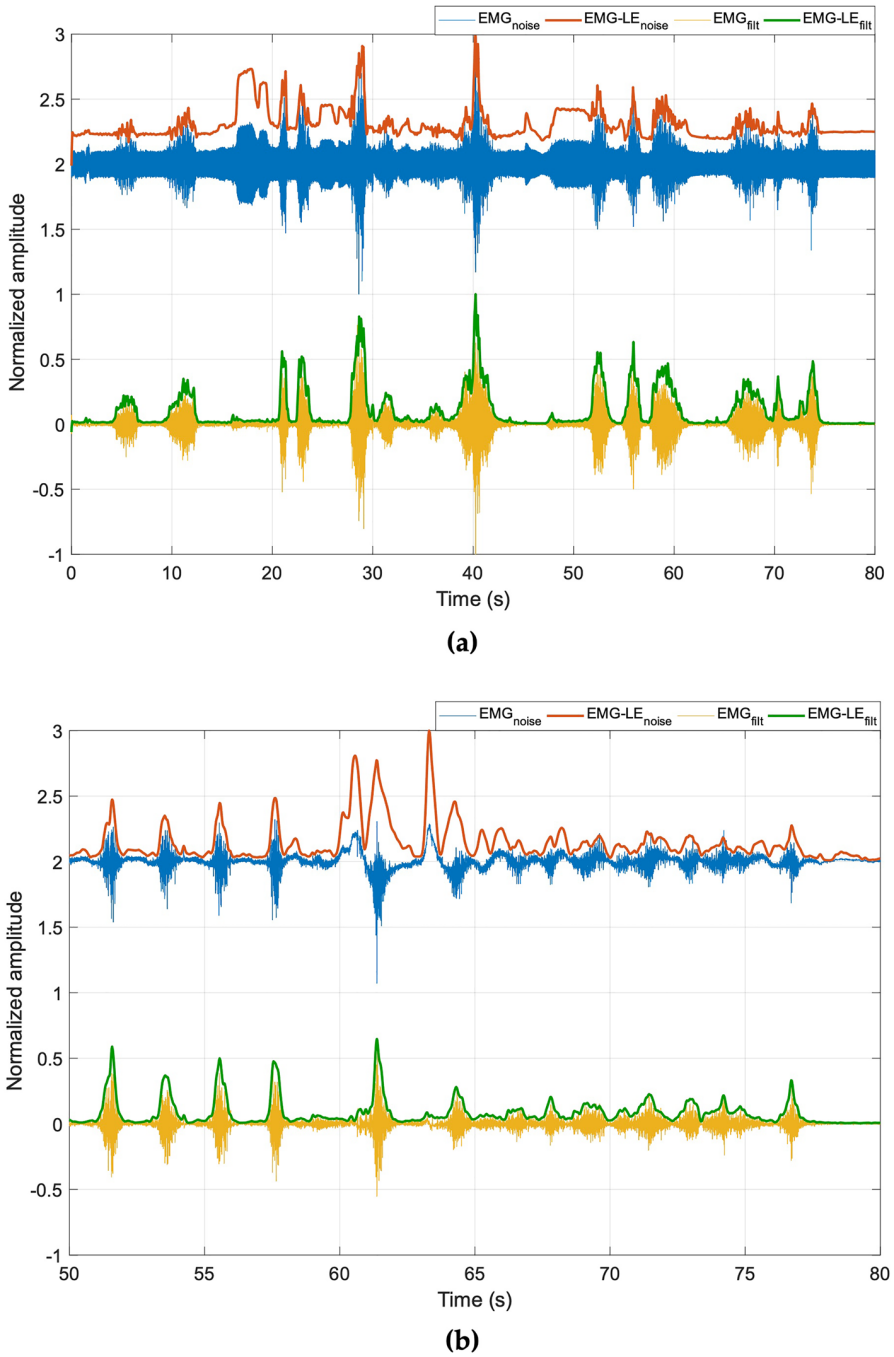
Results obtained on EMG corrupted by: (**a**) powerline interference; (**b**) motion artifacts. The noisy EMG is reported in blue, its linear envelope in red. The filtered EMG in yellow and its linear envelope in green.

Figure 9 shows the results achieved by the FFC filter on actual noisy EMG signals affected by amplitude modulated powerline interference (top panel) and motion artifacts (bottom panel). In Fig. 9a it could be observed how the amplitude variations of the powerline interference in the noisy EMG (in blue) resulted in spurious contraction peaks in the related EMG-LE (in red), and how they were effectively removed by the FFC filter in the processed EMG (in yellow) and the related EMG-LE (in green). Figure 9b demonstrates the capability of the FFC filter to remove the baseline oscillations due to motion artifacts, which likewise resulted in spurious contraction peaks in the noisy EMG-LE.

### Performance comparison with other denoising algorithms

The results obtained for the EMG signals corrupted by PLI with SNR equal to 0.05, both with constant and modulated amplitude, are outlined in Table 2. The Filter20x17 provided roughly a 1% increase in Pearson’s correlation coefficient between the filtered and the noiseless EMG-LE as compared to the FFC filter, while the FFT-based approach provided a slightly lower performance.

**Table 2:** Pearson’s correlation coefficients between the noiseless EMG-LE and the EMG-LE obtained by filtering noisy EMG signals with SNR = 0.05 via the considered algorithms.

Algorithm	PLI (constant amplitude)	PLI (modulated amplitude)
FFC	0.9857	0.9855
Filter20 × 17	0.9948	0.9946
FFT-based filter	0.9819	0.9817

**Figure 10:**
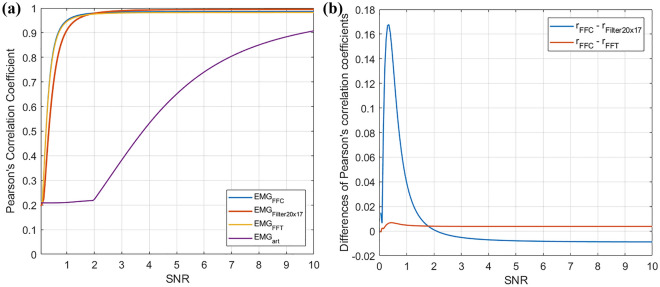
Results obtained on EMG corrupted by motion artifacts with SNR ranging from 0.05 to 10, and then processed via the FFC filter, the Filter20x17, and the FFT-based approach. (**a**) Pearson’s correlation coefficient between the noiseless and the filtered EMG-LE for the three compared algorithms; (**b**) differences between the correlation coefficients achieved via the FFC filter and those achieve via the Filter20x17 and the FFT-based approach.

The results obtained for EMG signals corrupted by motion artifacts (SNR ranging from 0.05 to 10) and processed via the compared algorithms are shown in Fig. 10. In particular, Fig. 10a depicts the Pearson’s correlation coefficients computed between the filtered EMG-LE signals and the noiseless EMG-LE reference, while Fig. 10b depicts the differences between the correlation coefficients obtained via the FFC algorithm and those obtained via the Filter20x17 and the FFT-based approach. The results show that for lower SNR values (SNR < 2) the FFC substantially outperforms the Filter20x17, by providing up to 35% increases in correlation coefficients, while for higher SNR values (SNR > 2) the Filter20x17 provides slight increases (< 1 %) in correlation coefficients with respect to the FFC filter. The FFT-based approach provides slightly lower performance as compared to the FFC for all SNR values.

The computational and memory resource requirements of the analyzed algorithms were outlined in Table 3, from which one can observe that the FFC filter features both the lowest computational burden, requiring the computation of only one sum and no multiplications, and the lowest memory storage of 20 input signal samples. The Filter20x17 requires the highest memory storage of 321 input signal samples, while the FFT-based filter presents the highest computational burden of at least 106 additions and 106 multiplications.

**Table 3:** Computational and memory resource requirements of the compared algorithms.

Algorithm	Additions	Multiplications	Divisions	Memory storage (samples)
FFC	1	0	0	20
Filter20 × 17	17	0	1	321
FFT-based filter	> 106	> 106	0	40

## Discussion

The algorithm presented in this study has the significant advantage of removing both powerline interference and motion artifacts on sEMG recordings in real time, as well as providing the EMG linear envelope with an extremely low computational load. It can be used to remove both 50 or 60 Hz PLI (and higher harmonics) by changing only the sampling frequency of the raw EMG signal. In addition, once the raw EMG signal is digitized and the FFC filter is applied, the EMG-LE is calculated using a rectification and a moving average low-pass filter.

Different tests were performed on EMG signals, artificially corrupted by powerline interference and motion artifacts, and proved the capacity of the FFC filter to effectively remove such noises. The proposed filter was capable of providing extremely accurate estimates of the noiseless EMG envelopes. Indeed, the Pearson’s correlation coefficients computed between the filtered and the true linear envelopes were always greater than 0.98, even for signals corrupted by PLI with SNR values as low as 0.05. Similar results were obtained for EMG signals corrupted by motion artifacts, with correlation coefficients in excess of 0.94, even for SNR values low as 1.

The FFC filter was further compared with two approaches previously proposed in literature, namely FilterDxN and an FFT-based approach, both in terms of quality of the filtered EMG-LE signals obtained, and in terms of computational and memory resource requirements. The results showed that both the FilterDxN and the FFT-based approach have substantially higher computational and memory resource requirements as compared to the FFC filter, which, in addition, always outperforms the FFT-based approach in terms of filtering efficacy, and also outperforms the FilterDxN on EMG signals that are heavily corrupted by motion artifacts while providing comparable performances for higher SNRs and for PLI removal. For these reasons, the FFC filter appears to be preferrable over the compared approaches for the purpose of reliable EMG-LE extraction, as it ensures, at the same time, substantially improved filtering efficacy and computational efficiency.

The proposed method provided excellent results also on real noisy EMG signals. Considering that the MyoWare board does not filter out the noises, its analog EMG-LE output would have provided very inaccurate estimates of muscle activity (see Fig. 3). This largely explains the practical problems encountered by many users of EMG sensor boards. The real-time performance of the FFC filter was confirmed by the effective implementation on the Arduino UNO board. Indeed, the design of a processing scheme with no multiplications and a rather small number of sums allowed its implementation on a simple and low-cost microcontroller like the Atmel ATmega328 equipped on the Arduino UNO board.

It is worth mentioning that the proposed filter cannot be used as a noise suppressor for other biopotentials such as electroencephalogram (EEG) and electrocardiogram (ECG). Indeed, the fundamental components of these signals would be unavoidably cancelled or distorted due to the high-pass cut-off, which is at about 12 Hz for 50 Hz PLI (about 15 Hz for 60 Hz PLI). The use of the proposed filter is also not recommended for the acquisition of EMG signals for diagnostic purposes. Although the high-pass cut-off at 12 Hz is recommended in many EMG applications, the FFC filter exhibits stop-bands at multiples of the powerline frequency that are too wide and cause distortions of the EMG signal^14^. This behaviour does not ensure that the FFC filter sufficiently preserves morphology of EMG signal components (i.e. motor unit action potential trains); nonetheless, this study showed that the FFC filter almost perfectly preserves the envelope of EMG signals. Moreover, the FFC filter is expected to provide lower performances in the removal of particularly brisk motion artifacts, as they would present with significant spectral power densities within the pass-bands of the filter. The performance of the FFC filter should be assessed on a larger cohort of cases, which may include EMG signals acquired from more subjects, in different conditions (e.g., fatigue), and performing different physical activities (e.g., running, playing sports).

As shown in a recent survey^2^, EMG-based HMIs are the most popular among those that exploit biopotentials as control signals. However, EMG recording presents well-known issues, such as need for stable electrode placement and skin preparation, degradation of wet electrodes contact when conductive gel dries out, degraded SNR that results from the use of dry electrodes^12^, susceptibility to electromagnetic interferences (e.g., PLI), motion artifacts, crosstalk with other biopotentials (e.g., EMG signals from near muscles, ECG, etc.). A further common issue of EMG is that electrodes applied to the skin expose the subjects to a higher risk of electrical shock^6,32–34^. This risk could be removed/reduced by using battery-operated devices and wireless connections^35^.

It is useful to highlight that, in order to overcome many of the EMG limitations, alternative methods such as Force-myography (FMG) are becoming increasingly popular to monitor muscle activity^2,36^ and control HMIs (e.g., hand prostheses^37–40^, exoskeletons^41^, gesture recognition devices^42^). FMG is currently the best alternative to EMG linear envelope^43^ exploits morphological changes in muscles during contraction by using force/pressure sensors, which have also been useful to detect respiration and mechanical heart activity^44–46^. Moreover, the FMG presents various advantages over the EMG: no need for electrodes, i.e. no electrical risk; much simpler processing, since the FMG signal can be used as it is and has a strong similarity with EMG-LE^47^; less susceptibility to electromagnetic interference. However, while well-established standards for EMG measurements are available (e.g., SENIAM^7^), clear guidelines to achieve accurate FMG measurements on specific muscles have not been provided yet.

It is interesting to note that integration of EMG and FMG improves classification of human intent and control of powered prosthetic devices, as compared to EMG alone^48^. Such integration would require an increased computational demand, because both EMG and FMG signals would need to be processed. The extremely low computational burden of the FFC filter would certainly help to reduce the computational burden of reliable EMG-LE extraction, thus favoring the development of these new kind of HMIs on very resource-constrained platforms developed for low-power applications.

In conclusion, although new emerging techniques are becoming more and more widespread and show comparable or even superior performances compared to EMG, this technique is still the current gold standard to monitor muscle activity^4,14^. Consequently, it is still useful to investigate new EMG signal filtering techniques that can increase its performance, especially in HMI applications^14,49–51^.

## Supplementary Information

Supplementary Information.

## Data Availability

The data presented in this study are available on request from the corresponding author.
