# Supplementary material for: A smart approach to EMG envelope extraction and powerful denoising for human–machine interfaces
Source: Sci Rep. 2023 May 12;13:7768. doi: 10.1038/s41598-023-33319-4 (PMC10181995; doi:10.1038/s41598-023-33319-4)
Supplement: Supplementary file 1 — Supplementary Information. [file 41598_2023_33319_MOESM1_ESM.pdf]

A Smart Approach to EMG Envelope Extraction and Powerful Denoising for Human-Machine Interfaces

Daniele Esposito[1], Jessica Centracchio[1,*], Paolo Bifulco[1], and Emilio Andreozzi[1]

[1] Department of Electrical Engineering and Information Technologies, University of Naples Federico II, Via Claudio, 21 80125 Napoli, Italy; daniele.esposito@unina.it; jessica.centracchio@unina.it; paolo.bifulco@unina.it; emilio.andreozzi@unina.it

*Corresponding author: jessica.centracchio@unina.it

Content of the supplementary material

This supplementary material describes the hardware setups and the Arduino codes for EMG signal acquisition and processing via methods proposed in the article, by using a "Myoware Muscle Sensor" and an Arduino UNO board.

1 Hardware setups

Two hardware setups are described, depending on the communication protocol to be used to send the data from the Arduino UNO board to the computer. The user can choose between an USB cable communication, and a Bluetooth serial communication.

In both setups, it is important to connect the “Raw EMG” output pin of the “Myoware Muscle Sensor”, and NOT the “SIGNAL” pin, to the analog input of the Arduino UNO board.

1.1. USB serial communication

Hardware requirements:

- Myoware Muscle Sensor
- Arduino UNO board
- USB cable
- Computer with USB port

Hardware connections:

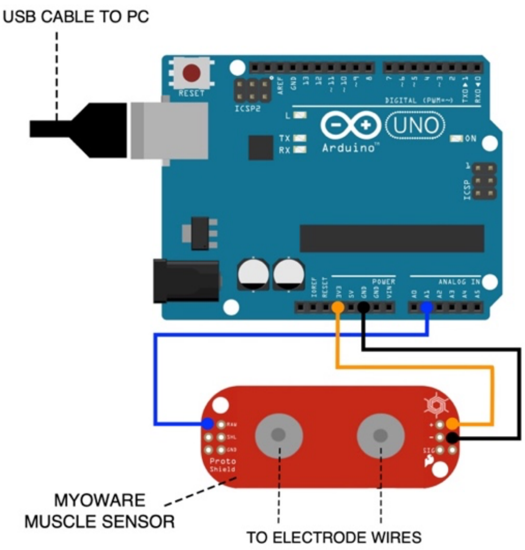

Figure S1. USB serial communication mode: schematic of the connections between the Arduino UNO board and the Myoware Muscle Sensor.

Table S1. USB serial communication mode: pin pairing between the Arduino UNO board and the Myoware Muscle Sensor.

**Arduino UNO**	**Myoware Muscle Sensor**
**A1** (Analog input 1)	**RAW** (raw EMG signal)
**3V3** (Voltage output 3.3V)	+ (Voltage input)
**GND** (Ground)	– (Ground)

Notes:

1. In addition to the two on-board electrode connectors, the “*Myoware Muscle Sensor*” features a third electrode connector for the reference electrode, which is not represented in the schematic. For correct EMG acquisition it is instrumental to connect a reference electrode to the “*Myoware Muscle Sensor*”. For more detailed information, please refer to the user manual of the “*Myoware Muscle Sensor*”.
2. When using a wired USB connection to a computer, it is very important to verify that the computer is either battery-powered, and not connected to a power outlet, because this could expose the subject connected to the EMG electrodes to a higher risk of electrical shock.

1.2. Bluetooth serial communication

Hardware requirements:

- Myoware Muscle Sensor
- Arduino UNO board
- HC-05 Bluetooth module
- Computer with Bluetooth connection

Hardware connections:

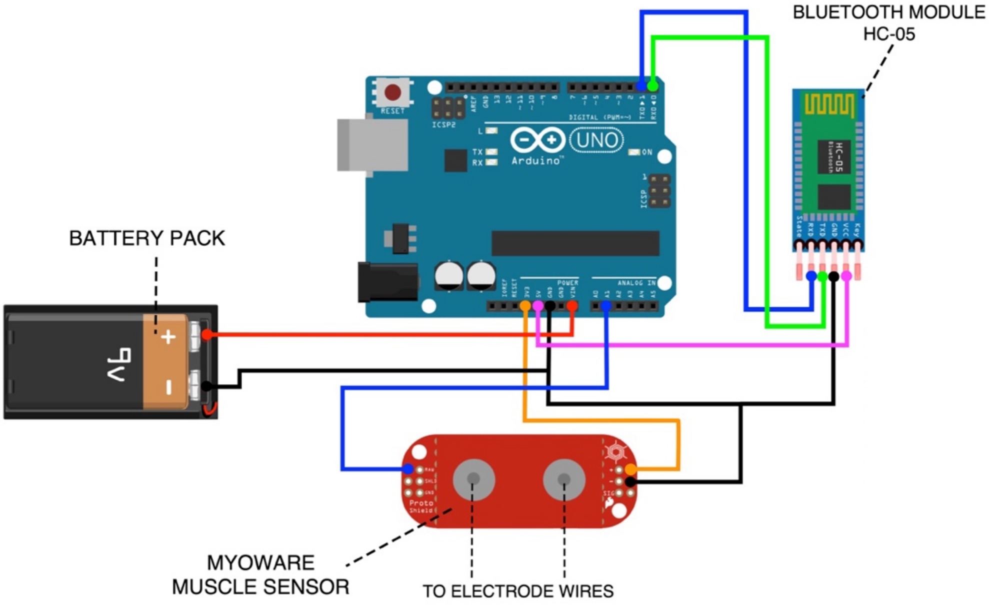

Figure S2. Bluetooth serial communication mode: schematic of the connections between the Arduino UNO board and the battery pack, the Myoware Muscle Sensor, and the HC-05 Bluetooth module.

Table S2. Bluetooth serial communication mode: pin pairing between the Arduino UNO board and the battery pack, the Myoware Muscle Sensor, and the HC-05 Bluetooth module.

Arduino UNO	Battery Pack	Myoware Muscle Sensor	HC-05 Bluetooth module
**Vin** (Voltage input)	Positive pole		
**GND** (Ground)	Negative pole		
**A1** (Analog input 1)		**RAW** (raw EMG signal)	
**3V3** (Voltage output 3.3V)		**+** (Voltage input)	
**GND** (Ground)		**–** (Ground)	
**5V** (Voltage output 5V)			**VCC** (Voltage input)
**GND** (Ground)			**GND** (Ground)
**1 – TXD** (sender)			**RXD** (receiver)
**0 – RXD** (receiver)			**TXD** (sender)

Notes:

1. In addition to the two on-board electrode connectors, the "*Myoware Muscle Sensor*" features a third electrode connector for the reference electrode, which is not represented in the schematic. For correct EMG acquisition it is instrumental to connect a reference electrode to the "*Myoware Muscle Sensor*". For more detailed information, please refer to the user manual of the "*Myoware Muscle Sensor*".

2 Arduino codes

2.1. USB serial communication

Software requirements:

- Arduino IDE application
- "TimerOne" library for Arduino (available for download at: https://www.arduinolibraries.info/libraries/timer-one (accessed on 18 January 2023)

Arduino code:

```
/************************************START********************** *******************/

// use TimerOne library in order to ensure a precise sampling frequency
#include <TimerOne.h>

// Declare global variables
unsigned int clk=0; //initialize clock
unsigned int k=0; // initialize counter
int sample=0; //initialize acquired sample
int last_sample=512; // set last sample
int ds=0; //inizialize the difference between two successive samples
long int S=0; // initialize the sum of the Moving Average
unsigned int win_len=128; // window length for computing EMG -LE (power of 2)
unsigned int EMG_level=0; // initialize EMG activity level
long ACC=0L; //initialize accumulator

// array of 20 elements to implement a Moving Average filter of 20 coefficients
int MAv[20]={0, 0, 0, 0, 0, 0, 0, 0, 0, 0, 0, 0, 0, 0, 0, 0, 0, 0, 0, 0};

/*****************************************************************************************
Function "EMG_LE" for:
1) sampling the raw EMG signal provided by the EMG sensor (e.g., MyoWare Muscle Sensor);
2) applying the feed-forward comb (FFC) filter (for removing powerline interference and motion artifacts) 3)
computing the EMG-LE
The function is triggered by the timer interrupt.
*****************************************************************************************/

void EMG_LE(){

// read current EMG raw signal
sample=analogRead(A1);

// compute the difference between two successive samples
ds=sample-last_sample;

// update Moving Average summation
S=S-MAv[k];
MAv[k]=ds;
S=S+MAv[k];

// update Moving Average counter
if (k<19){ k=k+1; }
else { k=0; }

// update accumulator
ACC=ACC+abs(S);
// update last_sample
last_sample=sample;
// update clock
```

```
clk=clk+1;

/*********************************************************************************************
EMG-LE is computed by applying a moving average with a window of 128 samples on the absolute value of the
EMG filtered by the FFC filter.
Division by 128 (2^7) is performed by shifting the bit string (representing the EMG_level variable) 7 positions
to the right.
*********************************************************************************************/
if (clk>=win_len)
        { EMG_level=ACC>>7;
        //reset accumulator and clock
        ACC=0;
        clk=0;
        }
/*********************************************************************************************
send on the serial port the EMG-LE signal to be used for Human Machine Interfaces control
*********************************************************************************************/
Serial.write(EMG_level);
}
/*********************************************************************************************
SETUP
* * * * **** **** ***** ***** **** ***** ***** **** ***** ***** **** **** ***** **/
void setup(){

// initialize the serial port to 115200 bps
Serial.begin(115200);

//connect RAW output of EMG sensor to the analog input - pin A1
pinMode(A1, INPUT);

/*********************************************************************************************
For removing 50Hz and higher armonics noise -> set the sampling frequency to 1000Hz -> DT=1ms -> activate
interrupt on timer1 every 1000us
For removing 60Hz and higher armonics noise -> set the sampling frequency to 1200Hz -> DT=0.833ms ->
activate interrupt on timer1 every 833us
*********************************************************************************************/
Timer1.initialize(1000);

// every time there is interrupt on timer1 call the function "EMG_LE"
Timer1.attachInterrupt(EMG_LE);
}
/*********************************************************************************************
LOOP
*********************************************************************************************/

void loop(){
        while (1) {}
        };

/***********************************END***************************************************/
```

Notes:

1. To access the data sent by the Arduino UNO board to the computer via the USB serial communication, a serial monitor application must be available on the computer, where the user must select the serial port number associated to the Arduino UNO board. The Arduino IDE offers a built-in serial monitor, which could be used for the purpose.
2. In the serial monitor application, it is important to choose the same baud rate specified in the Arduino code (115200). A different baud rate can be used by modifying the value specified in the Arduino code.

2.2. Bluetooth serial communication

Software requirements:

- Arduino IDE application
- "TimerOne" library for Arduino (available for download at: https://www.arduinolibraries.info/libraries/timer-one (accessed on 18 January 2023)

Arduino code:

```
/*************************************START*********************** *******************/
// use TimerOne library in order to ensure a precise sampling frequency
#include <TimerOne.h>

# use SoftwareSerial library for Bluetooth communication
include <SoftwareSerial.h>
SoftwareSerial bluetooth(rxPin, txPin);

// Declare global variables
int rxPin = 0; // HC-05 TXD must be connected to pin 0 (Arduino RXD)
int txPin = 1; // HC05 RXD must be connected to pin 1 (Arduino TXD)
unsigned int clk=0; //initialize clock
unsigned int k=0; // initialize counter
int sample=0; //initialize acquired sample
int last_sample=512; // set last sample
int ds=0; //inizialize the difference between two successive samples
long int S=0; // initialize the sum of the Moving Average
unsigned int win_len=128; // window length for computing EMG -LE (power of 2)
unsigned int EMG_level=0; // initialize EMG activity level
long ACC=0L; //initialize accumulator

// array of 20 elements to implement a Moving Average filter of 20 coefficients
int MAv[20]={0, 0, 0, 0, 0, 0, 0, 0, 0, 0, 0, 0, 0, 0, 0, 0, 0, 0, 0, 0};

/**************************************************************************************
Function "EMG_LE" for:
1) sampling the raw EMG signal provided by the EMG sensor (e.g., MyoWare Muscle Sensor);
2) applying the feed-forward comb (FFC) filter (for removing powerline interference and motion artifacts) 3)
computing the EMG-LE
The function is triggered by the timer interrupt.
**************************************************************************************/

void EMG_LE(){

// read current EMG raw signal
sample=analogRead(A1);

// compute the difference between two successive samples
ds=sample-last_sample;

// update Moving Average summation
S=S-MAv[k];
MAv[k]=ds;
S=S+MAv[k];

// update Moving Average counter
if (k<19){ k=k+1; }
else { k=0; }
// update accumulator
ACC=ACC+abs(S);
```

```
// update last_sample
last_sample=sample;
// update clock
clk=clk+1;

/*****************************************************************************************
EMG-LE is computed by applying a moving average with a window of 128 samples on the absolute value of the EMG filtered by the FFC filter.
Division by 128 (2^7) is performed by shifting the bit string (representing the EMG_level variable) 7 positions to the right.
*****************************************************************************************/
if (clk>=win_len)
        { EMG_level=ACC>>7;
        //reset accumulator and clock
        ACC=0;
        clk=0;
        }

/*****************************************************************************************
send on the serial port the EMG-LE signal to be used for Human Machine Interfaces control
*****************************************************************************************/
Serial.write(EMG_level);
}

/*****************************************************************************************
SETUP
* * * * **** **** ***** ***** **** ***** ***** **** ***** ***** **** **** ***** **/
void setup(){

// initialize the serial port to 9600 bps
Serial.begin(9600);
bluetooth.begin(9600); //set baud rate of Bluetooth serial communication

//connect RAW output of EMG sensor to the analog input - pin A1
pinMode(A1, INPUT);

/*****************************************************************************************
For removing 50Hz and higher armonics noise -> set the sampling frequency to 1000Hz -> DT=1ms -> activate interrupt on timer1 every 1000us
For removing 60Hz and higher armonics noise -> set the sampling frequency to 1200Hz -> DT=0.833ms -> activate interrupt on timer1 every 833us
*****************************************************************************************/

Timer1.initialize(1000);
// every time there is interrupt on timer1 call the function "EMG_LE"
Timer1.attachInterrupt(EMG_LE);
}

/*****************************************************************************************
LOOP
*****************************************************************************************/
void loop(){
        while (1) {}
        };
/***********************************END*************************************************/
```

Notes:

1. To access the data sent by the Arduino UNO board to the computer via the Bluetooth serial communication, a serial monitor application must be available on the computer, where the user must select the serial port number associated to the HC-05 Bluetooth transceiver.
2. In the serial monitor application, it is important to choose the same baud rate specified in the Arduino code (9600). A different baud rate can be used by modifying the value specified in the Arduino code.
